# Open questions in autoimmunity: discussions from the 2013 Controversies in Rheumatology and Autoimmunity Meeting

**DOI:** 10.1186/1741-7015-12-50

**Published:** 2014-03-18

**Authors:** Carlo Selmi, Yehuda Shoenfeld

**Affiliations:** 1 Division of Rheumatology, Allergy, and Clinical Immunology, University of California, Davis, USA; 2 Division of Rheumatology and Clinical Immunology, Humanitas Clinical and Research Center, Milan, Italy; 3 Zabludowicz Center for Autoimmune Diseases, Sheba Medical Center, Tel-Hashomer 52621, Israel Incumbent of the Laura Schwarz-Kipp Chair for Research of Autoimmune Diseases, Sackler Faculty of Medicine, Tel-Aviv University, Israel

**Keywords:** Anti-nuclear antibodies, Atherosclerosis, Cardiovascular system, DNA methylation, microRNA, Rheumatoid arthritis, Ultrasonography

## Abstract

The recent CORA (Controversies in Rheumatology and Autoimmunity) meeting held in 2013 represented a unique opportunity for rheumatologists to address several topics. Among these, four topics include: (i) the role of epigenetic changes in the pathogenesis of rheumatoid arthritis (RA), as shown by studies in monozygotic twins; (ii) the cardiovascular and atherosclerotic risk in patients with RA treated with biologics; (iii) the use of new biomarkers for the diagnosis and follow-up of RA and other autoimmune diseases, as represented by the new automatic machines for anti-nuclear antibodies detection, or ultrasound imaging to follow RA progression; and (iv) the latest guidelines on how to use and manage biologic therapies in RA and other autoimmune diseases, such as lupus. In summary, we will herein present these topics of discussion and underline the conclusions obtained by rheumatologists during the 2013 CORA Meeting.

## Introduction

In the last decade, autoimmune diseases have become a hot topic of discussion because of their increasing prevalence worldwide. Thanks to the new findings in laboratory and imaging techniques, it is now possible to achieve an earlier diagnosis and to start follow-up and therapies as soon as possible. However, despite significant improvements in these aspects, we still face many limitations in the management of autoimmune patients and in our previous contributions we have attempted to provide a picture of the current status of the hot topics that remain to be solved in the field of rheumatology and autoimmunity [1]. During the 2013 CORA (Controversies in Rheumatology and Autoimmunity) congress held in Budapest, several such issues were directly addressed by authoritative rheumatologists and are well illustrated by contributions recently published in *BMC Medicine*. These include: (i) the pathogenetic issues linked to epigenetics in rheumatoid arthritis (RA); (ii) the increased atherosclerotic and cardiovascular risk in RA patients treated with biologic therapy; (iii) the new laboratory and imaging biomarkers in RA and other connective tissue diseases; and (iv) the management of biologic therapies in RA and other autoimmune diseases.

### An epigenetic basis for RA

Numerous studies conducted in monozygotic (MZ) twins have demonstrated that in the majority of pairs only one of them will develop autoimmunity, despite the identical genome [2,3], and this appears to be secondary to environmental factors acting on an individual susceptibility through epigenetics. Epigenetic factors include DNA methylation, histone deacetylation and expression of non-coding small RNAs called microRNA [4] which cumulatively determine the cell-specific gene expression and, when altered, may lead to the onset of autoimmune diseases, as observed in systemic lupus erythematosus (SLE), Sjögren syndrome and scleroderma [4-11]. This paradigm applies also to RA, as discussed in the paper by Glant *et al.*[12]. The authors provide a comprehensive review of the most controversial issues in epigenetics studies, such as the importance of stating the specific cell type investigated in epigenetic studies. In fact, the epigenetic signature varies widely in different cell types, as the authors show when studying the DNA methylation status of RA synovial fibroblasts and peripheral blood mononuclear cells, similar to that observed elsewhere in effector cells [11,13]. These mechanisms seem to act on the same pathway able to control gene expression, the NFkB pathway, and, thus, influence the inflammatory and immune response.

The importance of studies on epigenetics relies on the possibility to use the results for diagnostic and therapeutic purposes. In fact, several trials are ongoing for the use of new therapies that alter the epigenetic signature in a specific disease, mainly in cancer, but no epigenetics-based drug is currently approved for clinical use [6].

### The cardiovascular and cancer risks in rheumatology

Whether there is a higher risk of atherosclerosis and cardiovascular events as well as cancer in patients with RA and other autoimmune diseases is an issue of enormous importance as the diagnosis of a rheumatological condition is made at increasingly young ages [14-16]. It is well established that autoimmune diseases are characterized by a chronic state of systemic inflammation [17,18], mediated also by microRNA [19-22] and by the production of autoantibodies that can induce a higher risk of thromboembolism and atherosclerosis compared to the general population [23,24], as observed in the antiphospholipid syndrome [14,25]. Matsuura *et al.* describes the main mechanisms that trigger inflammation in the atherosclerotic plaque, leading to its rupture and to the cardiovascular event [26]. Among these factors, a key role is played by pro-inflammatory cytokines (that is, interleukin-1 beta (IL-1β)) and caspases that activate the NLRP3 inflammasome together with lysosomal damage and reactive oxygen species. Of note, this unsuspected connection strengthens the current view that autoimmunity and autoinflammatory diseases are indeed linked in their pathogenetic mechanisms [27-29]. The prolonged inflammatory state further induces the production of C-reactive protein (CRP) that is capable of binding oxidized low density lipoprotein (oxLDL) and this complex induces an alteration of the arterial wall that accelerates atherosclerosis.

An antigenic component that plays a pathogenic role in autoimmune diseases is β2GPI, well known as the target of anti- β2GPI antibodies in the anti-phospholipid syndrome [17,30-33]. Also, this antigen can bind oxLDL and lead to the perpetuation of the inflammatory state in the vascular wall [17]. The two diseases considered by Matsuura and colleagues as prototypes of chronic inflammation are RA and SLE which are associated with an increased cardiovascular risk and because they have high levels of anti-oxLDL and anti-oxLDL/β2GPI antibodies. Also, anti-nuclear antibodies (ANA) are often observed in autoimmune diseases [34,35] and a multivariate analysis showed that ANA are inversely correlated with carotid elasticity. From a cellular point of view, Th17 and Treg cells also play an important role in atherogenesis, which is protective and anti-atherogenic for Tregs and is still unknown for Th17. In conclusion, the authors describe all the inflammatory and autoimmune elements that induce higher risk of atherosclerosis in autoimmune diseases [26]. Damjanov *et al.* tackle the topic from a different point of view and address a key question which is complementary to the former, that is, the effect of anti-tumor necrosis factor alpha (TNFα) therapy on the cardiovascular risk in RA patients [36]. The discussion of this topic shows that biologic therapies reduce the cardiovascular risk in RA, as supported also by previous reports. However, we remain unaware of the mechanisms by which anti-TNFα therapies can influence the inflammatory processes responsible for the altered vascular function and lipid profile that is transiently modified [37]. An additional element that Damjanov and colleagues consider when describing the link of biologic therapy and cardiovascular disease in autoimmunity is that these new therapies also seem to increase the risk of cancer, mainly represented by melanoma and non-melanoma skin cancer, despite a possibly enhanced risk associated with autoimmunity *per se*. In conclusion, biologic therapy reduces the cardiovascular risk in RA but may be responsible for increased risk of skin cancer. However, this risk should not be considered sufficient to induce changes in clinical practice or in treatment indication.

### New frontiers in the use of biologic therapies

The use of biologic therapies for the treatment of RA patients has completely changed the management and follow-up of these patients in the last 15 years. Several biologic drugs are now available, and they can be administered in different ways and with different timing, so that rheumatologists can tailor the biologic therapy according to what is better for each RA patient. However, basic questions still remain open, as discussed by Van Vollenhoven *et al.*[38] in this issue. The authors mainly discuss the possibility that an early start of biologic therapies can help in achieving remission of RA, and this can later lead to better outcome and early interruption of biologic therapies. Several trials are studying different therapeutic approaches to be able to induce remission in early RA patients who failed methotrexate monotherapy, but results are not clear yet. Another hot topic concerning biologic therapies is discussed in this issue by Gatto *et al.* who discuss the possibility to use biologic therapies off-label for patients with systemic lupus erythematosus (SLE) [39]. Several trials were started to test therapies such as rituximab, epratuzumab, belimumab, abatacept and anti-TNFα drugs in SLE patients, but only belimumab has been approved and is currently available for mild-to-moderate SLE patients. The reasons for the failure of the other drugs are several, from the extreme heterogeneity of SLE disease manifestations to the differences of the enrolled SLE population (that is, ethnic background, past medications) and study design. So the next question asked by Mocsai *et al.* is: what is the future of targeted therapy in rheumatology? [40]. The authors attempt to answer by considering the recent advances in the development not only of biologics but also of small molecules for rheumatic diseases, showing that these new therapies represent a big change in the management of rheumatic patients. Several trials are ongoing to test small-molecule anti-rheumatic agents, to identify the better administration route and the characteristics of the target patients. The authors expect wide development of new safe and effective biologics in the future and also oral therapies that could further improve the management of rheumatic patients.

### Diagnostic tools in rheumatology

Serum autoantibodies are the main laboratory biomarker for the diagnosis of systemic autoimmune rheumatic diseases and ANA identified by indirect immunofluorescence have high sensitivity and specificity rates, albeit remaining largely dependent on operator expertise. Meroni *et al.* describe the recent findings in automated ANA detection and the strengths and limitations of this approach in clinical practice [41], as discussed in a recent consensus study and extensively during the CORA congress. In fact, traditional indirect immunofluorescence is time and labor consuming, and is based on the recognition of specific patterns by a skilled operator. To overcome these limitations, new automated systems have been developed by several companies to allow the recognition of ANA positive or negative samples that can be further characterized by their specific pattern and titer [42,43]. The advantages of these automated systems are obvious and include the reduction of time and cost for indirect immunofluorescence (IIF), and the increased number of samples that can be reliably tested, but problems have been also identified [43]. For example, automated IIF for ANA detection fails to describe accurately mixed patterns, cytoplasmic staining or rare patterns. These limits can be a serious problem for the use of ANA in the diagnosis of autoimmune diseases such as scleroderma [44], a disease characterized by the presence of rare and complex ANA patterns at indirect immunofluorescence that cannot be easily identified by current automated systems [35,45]. Meroni and colleagues state that despite several advantages, automated ANA IIF assays need further study and improvement before replacing standard IIF for ANA detection [46].

Beside ANA, other autoantibodies are very important for the diagnosis of rheumatic diseases, as in the case of anti-citrullinated peptides antibodies (ACPA) in RA. In the present issue, Senolt *et al.*[47] describe the recent finding of new autoantibodies, the anti-carbamylated antigens (anti-CarP) [48], peptidyl arginine deiminase type 4 (PAD4) and v raf murine sarcoma viral oncogene homolog B1 (BRAF) [49,50]. Their role is to improve the early diagnosis of RA, along with the classical and most sensitive (albeit poorly specific) autoantibodies detected in RA, such as the rheumatoid factor. The authors describe these serological biomarkers to underline their predictive and prognostic value, and they also suggest a role in therapy monitoring through the autoantibody titer fluctuation, but this needs further investigation.

Ultrasound (US) imaging plays a growing role in RA management together with the serological biomarkers that were previously discussed. The same authors also illustrate the main US features that help in the diagnosis of RA [47]. In fact, US allows to identify fluid collection in the joint, synovial hypertrophy, cartilage damage, bone erosions, tendinopathy, enthesitis, and presence of a Doppler signal if hyperemia due to inflammation is present. The authors state that ultrasound can be currently considered as a reliable biomarker of synovitis, and in fact the EULAR/ACR 2010 classification criteria for RA included US as a means of confirmation of clinical findings of joint involvement.

## Conclusions

It should now be clear from our discussion that the most prominent issues in the management and understanding of rheumatological conditions warrant an extensive discussion to gather sufficient agreement. As such, we are convinced that opportunities such as the CORA meeting that took place in 2013 and the one planned in Sorrento on March 12-15, 2015 provide the ideal arena to address the remaining questions on the table. With the growing burden related to autoimmune diseases we are convinced, for example, that a shared approach to the eligibility of patients to expensive treatments or a better understanding of the true role of autoantibodies is to be encouraged among experts worldwide.

## Competing interests

The authors declare they have no competing interests.
